# Body composition in patients with schizophrenia: Comparison with healthy controls

**DOI:** 10.1186/1744-859X-11-11

**Published:** 2012-05-03

**Authors:** Norio Sugawara, Norio Yasui-Furukori, Shoko Tsuchimine, Akira Fujii, Yasushi Sato, Manabu Saito, Masashi Matsuzaka, Ippei Takahashi, Sunao Kaneko

**Affiliations:** 1 Department of Neuropsychiatry, Hirosaki University School of Medicine, Hirosaki, Japan; 2 Department of Social Medicine, Hirosaki University School of Medicine, Hirosaki, Japan; 3 Department of Psychiatry, Hirosaki-Aiseikai Hospital, Hirosaki, Japan

**Keywords:** Body composition, Bioelectrical impedance, Schizophrenia, Japanese

## Abstract

**Background:**

Recently, a relationship between obesity and schizophrenia has been reported. Although fat- mass and fat free mass have been shown to be more predictive of health risk than body mass index, there are limited findings about body composition among patients suffering from schizophrenia. The aim of this study is to compare the body composition of schizophrenia patients with that of healthy subjects in Japan.

**Methods:**

We recruited patients (n = 204), aged 41.3 ± 13.8 (mean ± SD) years old with the DSM-IV diagnosis of schizophrenia who were admitted to psychiatric hospital using a cross-sectional design. Subjects' anthropometric measurements including weight, height, body mass index (BMI), and medications were also collected. Body fat, percent (%) body fat, fat- free mass, muscle mass, and body water were measured using the bioelectrical impedance analysis (BIA) method. Comparative analysis was performed with schizophrenic subjects and 204 healthy control individuals.

**Results:**

In a multiple regression model with age, body mass index, and dose in chlorpromazine equivalents, schizophrenia was a significantly linked with more body fat, higher % body fat, lower fat- free mass, lower muscle mass, and lower body water among males. In females, schizophrenia had a significant association with lower % body fat, higher fat- free mass, higher muscle mass, and higher body water.

**Conclusions:**

Our data demonstrate gender differences with regard to changes in body composition in association with schizophrenia. These results indicate that intervention programs designed to fight obesity among schizophrenic patients should be individualized according to gender.

## Introduction

The prevalence of obesity among patients with schizophrenia is higher than the general population [1,2]. Obesity among patients with schizophrenia is a growing concern because being overweight is a major risk factor for metabolic syndrome, cardiovascular diseases, and premature death, and this risk is nearly double that of the general population [3-5]. In addition, in people with schizophrenia, obesity is associated with lower self-esteem, poorer psychosocial adaptation [6], reduced quality of life [7], non-compliance with antipsychotic medication regime [8] and increased medication cost [9].

In previous studies that have considered obesity in patients with schizophrenia, weight or body mass index (BMI) was commonly used as measurement parameters. However, it is important to consider body composition in detail because the excess accumulation of body fat, rather than BMI, causes the health risks associated with obesity [10]. Although BMI correlates highly with fat mass, depending on the level of muscularity, this value can be misleading regarding the level of adiposity of an individual.

Recently, a bioelectrical impedance analysis (BIA) method was developed that can measure body composition variables. BIA is based on the principle that there is less resistance to an alternating current passing through tissues that contain fluids and electrolytes than those containing relatively high amounts of lipids [11]. BIA results correlate well with the results of dual energy X-ray absorptiometry (DXA) [12,13]. BIA is a relatively quick procedure that is inexpensive and suitable for examining large numbers of people for studies.

The objectives of this study were to use BIA to compare the body composition variables between patients with schizophrenia and healthy individuals. To our knowledge, this is the largest study of this nature carried out in an Asian population.

## Methods

### Participants

This study was conducted between July 2010 and November 2010. The subjects were 204 outpatients (74 males and 130 females) at Hirosaki University Hospital in Japan who were diagnosed with either schizophrenia or schizoaffective disorder based on the DSM-IV diagnostic criteria. The diagnoses of the patients were recorded from their medical charts. As a reference group, 204 healthy volunteers (74 males and 130 females) were also included. The data collection for this study was approved by the Ethics Committee of the Hirosaki University School of Medicine and all subjects provided written informed consent before participating in this study.

### Procedure

Information on the subjects’ demographic data (age and sex), and current medications was obtained from their medical records. In order to have comparable value for each patient, medication was converted to chlorpromazine equivalents [14]. Patient heights were determined without shoes on a portable stadiometer with the mandible plane parallel to the floor. Body composition was measured using the Tanita MC-190 body composition analyzer [15] with correction for light indoor clothing. The measurement procedure required the subject to stand barefooted on the analyzer and to hold a pair of handgrips, one in each hand. The device uses multiple-frequency (5 kHz, 50 kHz, 250 kHz, and 500 kHz) BIA technology and has 8 tactile electrodes: 2 are in contact with the palm and thumb of each hand, and 2 are in contact with the anterior and posterior aspect of the sole of each foot. To calculate body fat, percent (%) body fat, fat- free mass, muscle mass, and body water for the entire body, the MC-190 uses a proprietary equation developed by the manufacturer.

### Statistical analysis

Descriptive statistical analyses were performed to describe the demographic and clinical variables. To compare the main demographic and clinical characteristics between patients and control, an unpaired Student’s t-test was performed to analyze continuous variables. Data are presented as means ± SD. Multiple linear regression analysis was employed to analyze the continuous variables of body composition. Healthy individuals were used as the reference category. Regression analyses were conducted in adjusted conditions for confounding factors (age, BMI, and dose in CP equivalents). A value of p < 0.05 was considered significant. The data were analyzed using the PASW Statistics-PC-software for Windows, Version 18.0.0.

## Results

### Demographic characteristics

The characteristics of participant are listed in Table 1. Among males, height was significantly higher in healthy individuals than in patients with schizophrenia. In females, height and body weight were significantly higher in patients with schizophrenia than in healthy individuals. Body mass index was significantly higher in patients with schizophrenia than in healthy individuals among both genders. No differences were observed for any of the other characteristics.

**Table 1:** Demographic characteristics of patients with schizophrenia and healthy controls

	**Male**			**Female**		
	**Schizophrenia**	**Control**	**p value**	**Schizophrenia**	**Control**	**p value**
Age (years old)	39.0±13.5	40.8±11.8	p=0.398	42.5±13.9	43.4±12.9	p=0.592
Height (cm)	165.5±8.6	170.8±6.5	p<0.001	160.6±7.9	157.6±6.1	p<0.01
Body weight (kg)	69.2±16.5	68.5±10.7	p=0.767	63.4±13.0	54.8±10.1	p<0.001
BMI (kg/m^2^)	25.2±5.5	23.4±3.2	p<0.05	24.6±5.0	22.1±4.0	p<0.001

### Body composition measurements

Table 2 shows the body composition measurements of patients with schizophrenia and healthy controls. Among males, body fat, and % body fat were significantly higher in patients with schizophrenia than in healthy individuals; hence, fat- free mass, muscle mass, and body water were significantly higher in healthy individuals than in patients with schizophrenia. In females, body fat, fat- free mass, muscle mass, and body water were significantly higher in patients with schizophrenia than in healthy individuals.

**Table 2:** Body composition measurements of patients with schizophrenia and healthy controls

	**Male**			**Female**		
	**Schizophrenia**	**Control**	**p value**	**Schizophrenia**	**Control**	**p value**
Body Fat (kg)	20.2±12.4	13.0±5.8	p<0.001	19.8±9.8	16.3±7.6	p<0.01
% Body Fat	27.7±11.8	18.3±5.8	p<0.001	30.2±9.8	28.5±7.6	p=0.124
Fat Free Mass (kg)	49.0±9.7	55.5±5.9	p<0.001	43.6±7.9	38.5±3.6	p<0.001
Muscle Mass (kg)	46.3±9.2	52.6±5.6	p<0.001	41.2±7.5	36.3±3.3	p<0.001
Body Water (kg)	34.3±6.1	38.4±4.6	p<0.001	31.3±5.2	27.8±3.3	p<0.001

### Multiple regression analysis for the detailed body composition characteristics

The results of the regression analyses examining the associations between the diagnosis of schizophrenia and body composition are presented in Table 3. Among males, schizophrenia was a significantly associated with higher body fat, higher % body fat, lower fat- free mass, lower muscle mass, and lower body water. In females, being schizophrenic had a significant association with lower % body fat, higher fat free mass, higher muscle mass, and higher body water.

**Table 3:** Multiple regression analysis for the detailed body composition

		**male**		**female**	
		**Beta coefficient**	**p-value**	**Beta coefficient**	**p-value**
Body Fat					
	Age	−0.094	p < 0.01	−0.023	p = 0.241
	BMI	0.882	p < 0.001	0.972	p < 0.001
	Schizophrenia	0.188	p < 0.001	−0.041	p = 0.105
	Dose in CP equivalents	−0.031	P = 0.402	−0.041	p = 0.113
% Body Fat					
	Age	−0.109	p < 0.05	0.100	p < 0.01
	BMI	0.735	p < 0.001	0.867	p < 0.001
	Schizophrenia	0.360	p < 0.001	−0.094	p < 0.05
	Dose in CP equivalents	−0.093	p = 0.097	−0.069	p = 0.108
					
	Age	0.022	p = 0.755	−0.238	p < 0.001
	BMI	0.369	p < 0.001	0.322	p < 0.001
	Schizophrenia	−0.531	p < 0.001	0.194	p < 0.01
	Dose in CP equivalents	0.131	p = 0.157	0.149	p < 0.05
Muscle Mass					
	Age	0.025	p = 0.724	−0.233	p < 0.001
	BMI	0.362	p < 0.001	0.312	p < 0.001
	Schizophrenia	−0.534	p < 0.001	0.198	p < 0.01
	Dose in CP equivalents	0.132	p = 0.155	0.149	p < 0.05
Body in CP equivalents		0.118	p = 0.192	0.131	p < 0.05

## Discussion

The present study on the body composition of patients diagnosed with schizophrenia is the largest study of this nature in an Asian population. Compared to the reference group, male patients with schizophrenia were found to have higher % body fat and lower muscle mass. In contrast, female patients with schizophrenia did not exhibit this association.

Other studies [16-18] have compared the body composition of patients with schizophrenia and healthy individuals. Nilsson et al. [16] reported that 28 patients with schizophrenia had higher % body fat and lower fat- free mass than healthy controls. Satoh et al. [17] performed a study involving 80 male schizophrenic patients and 64 healthy male individuals and showed that patients with schizophrenia had higher % body fat than healthy controls. Furthermore, Saarni et al. [18] reported that schizophrenia (n = 58) was significantly associated with a higher % body fat and lower fat- free mass after adjusting for age, gender, and BMI.

It is now well recognized that adipose tissue, especially in the abdominal region, is associated with the secretion of pro-inflammatory (e.g., interleukin- 6, and tumor necrosis factor- alpha) and catabolic (e.g., leptin, and retinol- binding protein 4) agents [19,20]. These molecules, in concert with the insulin resistance associated with abdominal obesity, contribute to a loss of muscle mass [19].

The mechanism for increased body fat among males has not been completely elucidated. However, patients with schizophrenia are at risk for developing obesity due to poor dietary habits, lower resting energy expenditure, lack of exercise or limited activity due to the negative symptoms of schizophrenia. Previous studies [21,22] have suggested an increased propensity for storing excess fat as visceral adiposity in schizophrenic patients. In addition, hypercortisolemia due to abnormalities of the hypothalamic- pituitary- adrenal (HPA) axis might also affect the development of central fat accumulation [23,24].

In contrast with male patients with schizophrenia, we did not find a higher % body fat or lower muscle mass among schizophrenic females relative to control females after multiple regression analysis. Previous studies comparing the body composition of patients with schizophrenia and healthy individuals analyzed a mixed subject pool of both genders or only male subjects. Therefore, we are reporting for the first time a comparison of the body composition of female schizophrenia patients and healthy individuals. One possible explanation for differences according to gender is that estrogen may affect the body composition. Animal studies also suggest that estrogen may play a role in the prevention of obesity [25]. Ovariectomized animals have increased adiposity compared with animals with intact ovaries [26]. Estrogen treatment in ovariectomized animals has been associated with significantly reduced adipose mass and adipocyte size. In addition, estrogen has also been shown to protect the muscle mass by stabilizing the muscle membrane [27]. Another possible explanation is that differences in physical exercise and socio-environmental factors might affect the body composition.

The current study also has some limitations. First, it was a cross-sectional study. It will be necessary to carry out a follow-up survey to clarify the reason for differences in the body composition between male patients and the reference group. We intend to follow-up this cohort prospectively in order to assess metabolic changes during the course of the illness and as a function of antipsychotic regimes. Second, patient recruitment was restricted to outpatients presenting to the hospital for a review of their health problems. No other population groups, such as children, adolescents or unmedicated patients, were included. Third, all possible parameters were not included in this study such as dietary habits, physical activity levels, the duration of illness and treatment, schizophrenic symptoms and medications. In particular, antipsychotic medications may be important factors. The use of first vs. second generation antipsychotics might also confound the results [28]. A stratified analysis by according to medication is needed in the future study.

## Conclusion

This study has shown that the male patients with schizophrenia had more body fat and a lower muscle mass than healthy individuals. Previous studies [29,30] have suggested that long-term programs that incorporate nutrition, exercise, and behavioral interventions can prevent weight gain among schizophrenic patients. The intervention program for obesity among schizophrenic patients should be prepared individually for each gender.

## Competing interests

The authors of this manuscript have no conflicts of interest to disclose as described by the Journal. The work performed here was funded by NV Organon, now part of MSD, Merck.

## Authors contribution

NS conceived the study, designed the study, conducted the statistical analysis, interpreted the data and wrote the initial draft of the manuscript. SK and NYF had full access to all of the data in the study and take responsibility for the integrity of the data and the accuracy of the data analysis. ST, AF and YS contributed to study design and assisted in drafting the manuscript. MS, MM and IT completed initial survey construction, recruitment of participants. All authors have approved the manuscript.
